# Supplementary material for: A versatile Agrobacterium-based plant transformation system for genetic engineering of diverse citrus cultivars
Source: Front Plant Sci. 2022 Oct 12;13:878335. doi: 10.3389/fpls.2022.878335 (PMC9597469; doi:10.3389/fpls.2022.878335)
Supplement: Supplementary Figure 1 — Comparison of one-year-old Carrizo Citrange non-transgenic (NT) and two independent transgenic lines with a heterologous gene of interest (T1 and T2). [file Image_1.pdf]

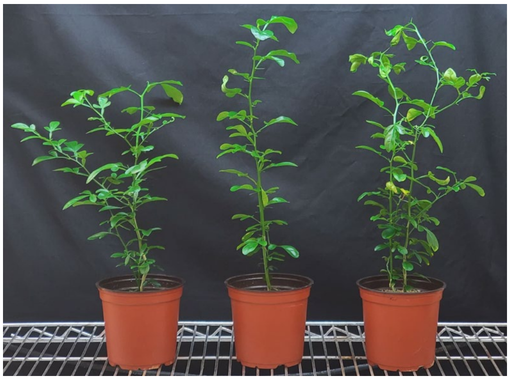

NT T1 T2

Supplementary Figure 1. Comparison of one-year-old Carrizo Citrange non-transgenic (NT) and two independent transgenic lines with a heterologous gene of interest (T1 and T2).
