# Supplementary material for: A versatile Agrobacterium-based plant transformation system for genetic engineering of diverse citrus cultivars
Source: Front Plant Sci. 2022 Oct 12;13:878335. doi: 10.3389/fpls.2022.878335 (PMC9597469; doi:10.3389/fpls.2022.878335)
Supplement: Supplementary Table 1 — Effects of pCH32 on transformation efficiency. Experiments were performed with atleast 10 replicates comprising ~22-25 explants each. [file Table_1.docx]

**Supplementary Table 1**. Effects of pCH32 on transformation efficiency. Experiments were performed with atleast 10 replicates comprising ~22-25 explants each.

| **Variety** | **Number of GUS Positive Shoots** | **Total Number of Explants** | **Transformation Efficiency**  **(%)** |
| --- | --- | --- | --- |
| Frost Lisbon – pCH32 | 4 | 236 | 1.7 |
| Frost Lisbon + pCH32 | 13 | 327 | 4.0 |
