# Supplementary material for: A versatile Agrobacterium-based plant transformation system for genetic engineering of diverse citrus cultivars
Source: Front Plant Sci. 2022 Oct 12;13:878335. doi: 10.3389/fpls.2022.878335 (PMC9597469; doi:10.3389/fpls.2022.878335)
Supplement: Supplementary Table 2 — Effects of A. tumefaciens density (OD600) on transformation efficiency. Experiments were performed with atleast 10 replicates comprising ~22-25 explants each. [file Table_2.docx]

**Supplementary Table 2**. Effects of *A. tumefaciens* density (OD_600_) on transformation efficiency. Experiments were performed with atleast 10 replicates comprising ~22-25 explants each.

| **Variety** | **OD_600_** | **Number of GUS Positive Shoots** | **Total Number of Explants** | **Transformation Efficiency**  **(%)** |
| --- | --- | --- | --- | --- |
| Frost Lisbon | 0.3 | 4 | 209 | 1.9 |
|  | 0.6 | 8 | 204 | 3.9 |
|  | 1.0 | 7 | 230 | 3.0 |
|  |  |  |  |  |
| Sour Orange | 0.3 | 8 | 420 | 1.9 |
|  | 0.6 | 6 | 264 | 2.3 |
|  | 1.0 | 0 | 502 | 0.0 |
|  |  |  |  |  |
| Mexican Lime | 0.3 | 22 | 1312 | 1.7 |
|  | 0.6 | 0 | 262 | 0.0 |
|  | 1 | 0 | 600 | 0.0 |
