# Supplementary material for: A versatile Agrobacterium-based plant transformation system for genetic engineering of diverse citrus cultivars
Source: Front Plant Sci. 2022 Oct 12;13:878335. doi: 10.3389/fpls.2022.878335 (PMC9597469; doi:10.3389/fpls.2022.878335)
Supplement: Supplementary Table 3 — Effects of Lipoic Acid (LA, 5 µM) on transformation efficiency. Experiments were performed with atleast 10 replicates comprising ~22-25 explants each. [file Table_3.docx]

**Supplementary Table 3**. Effects of Lipoic Acid (LA, 5 µM) on transformation efficiency. Experiments were performed with atleast 10 replicates comprising ~22-25 explants each.

| **Variety** | **Number of GUS Positive Shoots** | **Total Number of Explants** | **Transformation Efficiency**  **(%)** |
| --- | --- | --- | --- |
| Rio Red – LA | 6 | 495 | 1.2 |
| Rio Red + LA | 21 | 495 | 4.2 |
|  |  |  |  |
| Flying Dragon – LA | 3 | 300 | 1.0 |
| Flying Dragon + LA | 4 | 300 | 1.3 |
