# Supplementary material for: A versatile Agrobacterium-based plant transformation system for genetic engineering of diverse citrus cultivars
Source: Front Plant Sci. 2022 Oct 12;13:878335. doi: 10.3389/fpls.2022.878335 (PMC9597469; doi:10.3389/fpls.2022.878335)
Supplement: Supplementary Table 4 — Effects of Lipoic Acid (LA, 5 µM) and Spermidine (SPD, 1 mM) combination on transformation efficiency. Experiments were performed with atleast 10 replicates comprising ~22-25 explants each. [file Table_4.docx]

**Supplementary Table 4**. Effects of Lipoic Acid (LA, 5 µM) and Spermidine (SPD, 1 mM) combination on transformation efficiency. Experiments were performed with atleast 10 replicates comprising ~22-25 explants each.

| **Cultivar** | **Number of GUS Positive Shoots** | **Total Number of Explants** | **Transformation Efficiency**  **(%)** |
| --- | --- | --- | --- |
| Swingle + LA –SPD | 84 | 1300 | 6.5 |
| Swingle + LA +SPD | 73 | 1404 | 5.2 |
|  |  |  |  |
| Flying Dragon + LA –SPD | 12 | 360 | 3.3 |
| Flying Dragon + LA +SPD | 22 | 384 | 5.7 |
|  |  |  |  |
| Frost Lisbon + LA –SPD | 12 | 480 | 2.5 |
| Frost Lisbon + LA+SPD | 21 | 525 | 4.0 |
|  |  |  |  |
| Frost Eureka + LA –SPD | 3 | 300 | 1.0 |
| Frost Eureka + LA+SPD | 27 | 561 | 4.8 |
|  |  |  |  |
| Sweet orange + LA –SPD | 21 | 748 | 2.8 |
| Sweet orange + LA+SPD | 0 | 1594 | 0.0 |
